# Mesenchymal Stem Cells for Cardiac Regenerative Therapy: Optimization of Cell Differentiation Strategy

**DOI:** 10.1155/2015/524756

**Published:** 2015-08-03

**Authors:** Han Shen, Ying Wang, Zhiwei Zhang, Junjie Yang, Shijun Hu, Zhenya Shen

**Affiliations:** Department of Cardiovascular Surgery and Institute of Cardiovascular Science, First Affiliated Hospital of Soochow University, Suzhou, Jiangsu 215006, China

## Abstract

With the high mortality rate, coronary heart disease (CHD) has currently become a major life-threatening disease. The main pathological change of myocardial infarction (MI) is the induction of myocardial necrosis in infarction area which finally causes heart failure. Conventional treatments cannot regenerate the functional cell efficiently. Recent researches suggest that mesenchymal stem cells (MSCs) are able to differentiate into multiple lineages, including cardiomyocyte-like cells in vitro and in vivo, and they have been used for the treatment of MI to repair the injured myocardium and improve cardiac function. In this review, we will focus on the recent progress on MSCs derived cardiomyocytes for cardiac regeneration after MI.

## 1. Introduction

As the leading cause of mortality, cardiovascular disease is a major problem of global public health. Among cardiovascular diseases, coronary heart disease (CHD) is the main disease type causing the majority of deaths. At present, the treatment of CHD mainly includes medicine, percutaneous coronary intervention (PCI), and operation. To some extent, these treatments could improve myocardial ischemia and heart failure symptoms. Although the surgery operations make the occlusion artery unobstructed again, the damage to myocardial wall is irreversible. The current pharmacological and surgical measures are limited to palliative effects. Shortage in donor hearts and high cost are hindering the prevalence of heart transplantation. In 2001, Orlic et al. [1] transplanted autologous bone marrow mesenchymal stem cells (BMSCs) into mouse damaged heart and found these stem cells mostly differentiated into cardiomyocytes. This important discovery guided the scientists and clinicians to engage in plenty of researches on stem cells transplantation to treat myocardial infarction (MI). Significant progress has been made in the MSC research field, such as cell culture condition and technique of inducing differentiation in vitro [2, 3]. The differentiated myocardial cells from stem cells provide a promising perspective to cell treatment on cardiac diseases [4–6].

Stem cells include embryonic stem cells (ESCs) and adult stem cells (ASCs), commonly holding two major capabilities of self-renewal and differentiation. ASCs can be isolated from different adult tissues and can be differentiated into a variety of cell types [7]. As a kind of ASCs, mesenchymal stem cells (MSCs) have been described in nearly all postnatal tissues or organs, including umbilical cord blood [8, 9], placenta [10–12], and bone marrow [13], among others. MSCs represent an infrequent progenitor population with multiple differentiation potentials [14–19]. They are able to differentiate into several mesenchymal lineages, such as cartilage, muscle, vascular endothelial cells, and epidermic cells [20, 21]. With the advantage of autologous transplantation which avoids the immune rejection and ethical concerns, MSCs have great application prospect in personalized treatment of cardiovascular diseases [22–24].

## 2. The Induction Approaches of Cell Differentiation In Vitro and In Vivo

**Figure 1:**
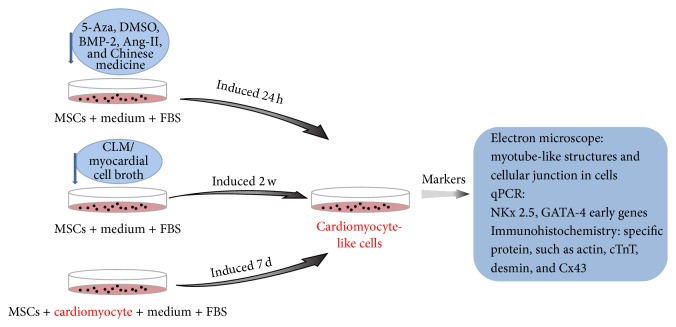
The diagram for the induction and identification of cardiomyocyte-like cells. MSCs cultured in medium supplemented with 5-Aza, DMSO, and BMP-2 will be induced to cardiomyocyte-like cells 24 h later. MSCs incubated in CLM/myocardial cell broth will differentiate to cardiomyocyte-like cells after 2 w. MSCs cocultured with cardiomyocyte will differentiated to cardiomyocyte-like cells 7 d later. The identification methods consist of morphology detection and molecular marker analysis.

Currently, the major methods to induce myocardial cell from BMSCs include biochemistry induction, myocardial microenvironment induction, and genetic modification (Figure 1).

### 2.1. Biochemical Substance

#### 2.1.1. 5-Azacytidine (5-Aza)

5-Aza, a chemical analogue of cytidine, is generally known as a demethylation pharmaceutical that can induce MSCs differentiation into cardiomyocyte-like cells by activating some dormant genes through demethylation [37]. In 1995, Wakitani et al. [25] first reported the successful isolation and culture of MSCs in vitro. After a 24-hour incubation with 5-Aza, they could observe myotube-like structures and cardiac-specific proteins expression in 7–10 d. These results showed that BMSCs could differentiate into cardiomyocyte-like cells with 5-Aza supplement, laying the foundation for BMSCs differentiation into cardiomyocyte-like cells. In 1999, Makino et al. [26] and others induced the immortalized BMSCs differentiation with 5-Aza. They observed myotube-like structures after 1 week, spontaneous beating after 2 weeks, and synchronous contraction after 3 weeks. The differentiated BMSCs not only expressed cardiac-specific proteins but also exhibited biological and electrophysiological characteristics of myocardial cells. Fukuda [38] found that the myocardial cells induced by 5-Aza had two kinds of action potentials. One comes from sinus nodal cells, and the other one might come from ventricular myocytes. Jaquet et al. [39] first separated human MSCs (hMSCs) for in vitro culture and incubated these hMSCs with 10 *μ*mol/L 5-Aza. The immunocytochemistry showed that 80% hMSCs expressed smooth muscle actin in two weeks, indicating these hMSCs might be differentiated into other muscle cells. Although 5-aza is the most commonly used chemical inducer, the differentiation efficiency is low, mainly due to the potential toxicity of 5-Aza and fat deposit in the cytoplasm which induce cell death. All the inducer applied in BMSCs differentiation are listed in Table 1.

#### 2.1.2. Bone Morphogenetic Protein-2 (BMP-2)

As a multifunctional glycoprotein, BMP-2 contributes to regulating of a wide variety of cell functions, including cell growth, differentiation, and apoptosis, among others [40]. Several studies have shown that the BMP-2 expression is initiated in early embryonic development [41]. BMP-2 plays a fundamental role in directed differentiation of cardiac stem cells and the development of embryonic heart through regulating the expressing of some cardiac transcription factors [42]. He Qizhi and Haijie [27] found that BMP-2 could also induce BMSCs differentiation into cardiomyocyte-like cells in vitro. Recent studies have shown that the roles of BMP-2 in gene expression of cardiogenic factors and cardiac differentiation from BMSCs were mediated by three molecular pathways: Smads, P38-MAPK, and PI-3K/Akt [43–45].

#### 2.1.3. Angiotensin-II (Ang-II)

Ang-II is capable of stimulating the proliferation of vascular smooth muscle cells [46] and fibroblast [47]. By regulating the signal of MAPK [48] and tumor growth factor (TGF) [49–51] and their consequent pathways, Ang-II can induce BMSCs to differentiate into cardiomyocyte-like cells. Xing et al. [28] induced BMSCs differentiation with Ang-II in vitro. After 4-week induction, the cells exhibited morphological characteristics of myocardial cells with cTnI expression and showed muscle wire-like structure under the electron microscope.

#### 2.1.4. DMSO

DMSO was proved to induce mouse P19 cells to differentiate into beating myocardial cells [29, 52–55]. DMSO plays a critical role in increasing the expression of prodynorphin and dynorphin B at the transcriptional level. It turns on both GATA4 and Nkx2.5 expressions, and then it recruits *α*-MHC and ventricle-specific cardiac myosin light chain-2 (MLC-2v) to form a functional compound [56]. Another study also showed that DMSO could mediate the releasing of calcium from intracellular stores in sarcoplasmic reticulum. Elevation of calcium concentration may play an important role in the induction of cell differentiation [57].

#### 2.1.5. Traditional Chinese Herb

Traditional Chinese herb can effectively induce stem cells differentiation into myocardial cells without any toxic or side effect [58]. Several studies [30, 59, 60] indicated that MSCs supplemented with by notoginsenoside in vitro could differentiate into cardiomyocyte-like cells. The morphologic features and characteristic markers of these cells were consistent with cardiomyocytes. Additional research [61] claimed that glucocorticoids released from myocardial tissue could induce BMSCs to migrate and differentiate into endothelial cells. There are several other traditional Chinese medicine inducers which also can drive MSCs to myocardial cells, such as Dan phenolic acid B [31], icariin [32], and astragaloside [33, 62].

### 2.2. Myocardial Microenvironment

#### 2.2.1. Myocardial Microenvironment In Vivo

Derived from the embryonic mesoderm, MSCs exhibit multiple differentiation potentials into mesoderm groups such as bones, cartilages, and myocardium under suitable conditions. Toma and his colleagues [34] reported that the transplanted hMSCs could successfully differentiate into myocardium and express myocardium specific proteins after cell transplantation into left ventricle of SCID mice. The myocardium specific proteins cTnT and phosphoprotein could regulate Ca-ATP activity in sarcoplasmic reticulum.

#### 2.2.2. Myocardial Microenvironment In Vitro

*(1) Cardiomyocyte Lysis Medium (CLM).* Yuan et al. [35] successfully initiated MSCs differentiation into cardiomyocyte-like cells using cardiac specific cell lysate, generated from primary myocardial cells. Cao et al. [63] induced hMSCs differentiation into cardiac myocytes with the minipig's cardiomyocyte lysate. These derived cardiomyocytes expressed cTnT, Cx43, and CD31. They also induced hMSCs differentiation with 5-Aza and differentiated cardiomyocytes expressed cTnT and Cx43, but not CD31. It is indicated that some compositions of CLM could also promote the differentiation from MSCs to endothelial cells which might help create basic conditions for revascularization.

*(2) The Supernatants of Cultured Cardiomyocytes.* Multiple evidence showed that BMSCs cultured in the media supplemented with myocardial cell culture supernatants could differentiate into cardiomyocyte-like cells [64]. Wang et al. [65] found that 10%, 20%, 30%, 40%, and 50% supernatants of the cardiomyocytes groups were used in induction of BMSCs, without morphological change. The expressions of a-SMA, *β*-actin, and troponin-T were significantly higher in 10%, 20%, 30%, 40%, and 50% supernatant of cardiomyocytes groups than those in control group, and the most significant percentage was 30%. Li et al. [66] found that the concentrations of insulin-like growth factor-1 (IGF-1), platelet-derived growth factor (PDGF), and fibroblast growth factor (FGF) in the supernatant of cardiomyocytes culture were significantly higher than that in BMSCs culture. Their results indicated that IGF-1, PDGF, and FGF in the supernatant of cardiomyocytes may have capability to induce BMSCs to differentiate into cardiomyocyte-like cells, and insulin-like growth factor may serve as the main cytokine.

*(3) Coculture with Myocardial Cells.* After coculturing GFP labeled rat MSCs with the cardiomyocytes in different proportions for 7 days, He et al. [67] successfully detected the cardiac specific proteins expression and action potential. Rangappa [36] and others indicated that the induction efficiency of MSCs cocultured with myocardial cells is obviously higher than those cultured alone. Through investigation on the structure of the gap conjunction between cardiomyocytes, Plotnikov and his colleagues claimed that direct contraction of cells was very important during the differentiation procedure [68–70].

### 2.3. Genetic Modification

In recent years, genetic modification has become a novel induction strategy which can converse BMSCs into myocardial cells in the molecular level. By inducing one or several key genes to activate cardiac gene networks, BMSCs could obtain cardiac differentiation. Several key transcription factors including Nkx2.5, GATA4, and TBX5 are expressed in the early cardiac development and regulate the expression of many cardiac structural proteins which are irreplaceable to the development of heart [71–75]. Recently, Jamali et al. found that exogenous Nkx2.5 gene expression could induce P19 cells to differentiate into cardiomyocyte-like cells alone [76]. Furthermore, with exogenous expression of Nkx2.5, the P19CL6 could differentiate into myocardial cells earlier and more efficiently when supplied with DMSO [77].

## 3. Identification of Successful Cardiac Differentiation from MSCs

BMSCs can differentiate into cardiomyocytes through the induction of chemicals, cytokine, and simulated cardiac microenvironment. The differentiated cells were polygonal or star-shape under the microscope. The ultrastructure and filament in the cytoplasm were observed by transmission microscope and cardiac specific cellular junction existing between cells.

First, we can detect the expression of cardiac marker genes Nkx 2.5 and GATA-4 by qPCR. Tissue-specific transcription factor GATA-4 and homologous nucleoprotein Nkx2.5 are two early markers of cardiac precursor cells, which play an important role in early cardiac development [78].

Second, we can test myocardial cell specific proteins including actin, cTnT, desmin, and Cx43 by immunofluorescence technique. Actin is the cytoskeletal proteins of the muscle cells, which is expressed in skeletal and cardiac muscle, and plays an important role in maintaining myofibrillar morphology and signal transmission in the sarcomere [79–81]. Cardiac troponin-T (cTnT) is only expressed in the myocardium, thus being a specific protein in the identification of myocardial cells [82, 83]. Desmin is the intermediate filament protein in muscle with 476 amino acids. It not only connects the adjacent myofibrils, but also connects myofibrils, nucleus, cytoskeleton, and organelle. Furthermore, desmin plays important roles in signal transduction [84]. Additional research [85] showed that desmin was involved in cell signal transduction and gene expression regulation which are closely related to left ventricular remodeling. Cx43 mainly exists in the atrial and ventricular muscle and participates in the formation of gap junctions. It composes three kinds of special structure of intercalated disc with intermediate junction and desmosome. Gap junctions mediate electrical and chemical coupling between adjacent cardiomyocytes, through forming the cell-to-cell pathways for orderly spread of the wave of electrical excitation responsible for a functional syncytium [86]. The expression of Cx43 in MSCs after induction indicates that myocardial cells own the morphological basis of the intercalated disc structure formation. It provides the material basis for the rhythmic systolic and diastolic motion. Cx43 maintains electrical activity and synchronization of systolic and diastolic functions which are very important to keep on myocardial function.

Adult cardiomyocytes show complicated electrophysiological characteristics. It has been shown that ion-channel proteins are expressed differently during differentiation. Two kinds of ion-channel proteins are expressed in early phase of sustained calcium current (Ica-L) and transient outward potassium current (Ito), but myocardial cells in later period of differentiation express all ion current: voltage dependent Na current (INa), delayed rectifier K current (Ik), inward rectifier K current (Ik1), muscarinic receptor agonist inward rectifier K current (IKAch), and the pacemaker current (If).

Additional research [2, 87–90] suggested that action potential consists of sinus node-like action potential, atrial muscle-like action potential, and ventricular-like action potential. These cells have the longer action potential duration and platform period, the smaller resting potential, and a pacemaker current slowly depolarizing in late diastole. The early cardiomyocytes express pacemaker-like cells action potential derived from two ion currents (Ica-L, Ik-to), whereas the late cardiomyocytes, such as the atrial and ventricular muscle cells, express three action potentials-a 75 mV resting potential, maximum action potential, and the overshoot rate [87].

## 4. MSCs-Based Clinical Therapy for MI

The most important aim of the basic researches of the MSCs is to serve the clinical treatment. MSCs, which have the ability to differentiate into cardiomyocyte-like cells, endothelial cells, and smooth muscle cells, become one of the most popular cells in MI treatment area. The cardiomyocyte-like cells can be differentiated from the MSCs in vitro with inducement of several external induction factors. These cardiomyocyte-like cells can be transplanted into MI patient and direct contact myocardial cells which provide a microenvironment of the induction of the MSCs into myocardial cells. As a result, it can help to repair the infracted heart muscles better.

### 4.1. Safety and Efficacy Evaluation of Stem-Cell Based Therapy

In 2001, the first case of the autologous stem cell transplantation for acute MI in clinical trials was carried out by Dr. Strauer who is a medical scientist from Dusseldorf of Germany [91]. A total of 1 × 10^7^ autologous stem cells were transplanted into infracted artery by catheter during percutaneous coronary angioplasty. After 10 weeks, it was shown that the intracoronary autologous stem cell transplantation for acute MI was safe and feasible through myocardial single photon emission computed tomography, echocardiogram, and nuclein ventriculography. At present, it is also verified that stem cell transplantation for ischemic heart disease treatment is safe and preliminary effective via clinical trials of REPAIR-AMI [92], MAGIC Cell-3-DES [93], BOOST [94], PROTECT-CAD [95], and so on.

### 4.2. The Suitable Transplant Time after MI

After MI, several factors are unfavorable for the survival of transplanted cells such as a large number of inflammatory cell infiltration, ischemical reperfusion injury, and microcirculatory disturbance. Meanwhile, a series of cell factors including stromal cell derived factor, vascular endothelial growth factor, and hepatocyte growth factor are upregulated, which is good for the aggregation, proliferation, and differentiation of transplanted cells toward the infarction area. Therefore, when to transplant is an important factor which affects the survival of transplanted cells and curative effect. If the transplantation is too early, a lot of transplanted cells will die due to the adverse local microenvironment. On the contrary, if it is too late, the transplanted effect is limited because of irreversible myocardial injury and formed ventricular remodeling.

There are several clinical trials carrying out the stem cell transplantation in different points of time. Comparing with the transplantation 1 hour after MI, the amount of survival cells are much less than that after 1-2 weeks, and the improvement of left ventricular function and reduction of the scar area is also lower [96]. The transplantation in 24 hours is not able to improve cardiac systolic function but can reduce the infarction area [97]. The research of REPAIR-AMI demonstrated that the BMSCs treatment in 4 days after MI is not beneficial but can improve cardiac systolic function in 4–8 days after infarction.

In 2009, MYSTAR trial first adopted the injection of autologous MNCs via both myocardial and coronary arteries to treat the MI patient. The LVEF of these patients was less than 45%. The transplant curative effect is measured by the differences between LVEF of early stage of AIM (3–6 weeks) and that of advanced stage (3-4 months).

### 4.3. The Dose of Transplant Cells after MI

The dosage of stem cells used to treat MI varied enormously between different investigations. In 2002, Ghostine et al. [98] injected 5 × 10^4^ cells by intramyocardial delivery system. Fukushima et al. [99] injected 5 × 10^6^ GFP-expressing skeletal myoblasts by either retrograde intracoronary or intramyocardial routes. As an urgent problem, researchers are pitching great effort in exploring the optimal transplanted cell dosage.

### 4.4. Delivery Route of Transplant Cells

#### 4.4.1. Intracoronary (IC) Artery Injection

MSCs are infused to injured sites by percutaneous artery injection into coronary artery. This approach ensures the higher dose of transplanted MSCs to infarction and its surrounding region at the first time [100]. In post-AMI study, this “homing” phenomenon about migration of cells into cardiomyocytes is only found in intracoronary injection instead of intravenous injection. This method is a common clinically practiced approach [101], but there are security issues. In patients with coronary artery obstructions, MSCs need to be infused by retrograde coronary venous (RCV) delivery system. Vicario et al. [102] and Yokoyama et al. [103] also provide correlated data in this area.

#### 4.4.2. Surgical Intramyocardial (IM) Injection

At present, most studies recommend transplanting stem cells by epicardial puncture under open-heart surgeries like CABG [104–106] or thoracoscopic. Intramyocardial injection has been the most accurate and direct approach for injecting stem cells to MI region of the heart. For its advantage of targeting localized myocardium, this method avoids many complex issues such as homing of the transplanted cells. The biggest drawback of IM injection is the invasive procedures, and the injection site is likely to cause cardiac arrhythmia and systemic embolization [107].

#### 4.4.3. Intravenous (IV) Infusion

Without heart surgery and catheterization, intravenous injection of stem cells is a simple and least invasive delivery route. In an experimental model of acute MI, heart function was improved significantly by peripheral intravenous injection of EPCs or BMSCs [108], but a lot of transplanted cells remained outside of the myocardium [109]. This limited the clinical application.

#### 4.4.4. Tissue Engineering Technology

Tissue engineering technology is a novel strategy to improve the efficacy of cell engraftment. MSCs are cultured on biological materials such as a hydrogel, 3D scaffold to form monolayer cells with better cell-to-cell adhesion. This enables direct tissue transplants and minimizes loss of cells [110]. The engrafted sheet survived on ischemic myocardium and grew to a thick stratum including some newly formed vessels and cardiomyocytes [111]. The technology creates an excellent environment which is suitable for MSCs survival, proliferation, and differentiation.

### 4.5. Assessment of Various Cell Delivery Methods

In order to detect the cell viability and repair effect of BMSCs delivered via different route, tracing technology and cardiac ultrasound are applied. Hou et al. [112] traced cells via radioactively labelling to evaluate the efficacy of cell engraftment. They reported 11 (surgical injection), 2.6 (coronary artery), and 3.2% (coronary venous) of them being retained, respectively. Lee et al. [113] dual labeled the stem cells with HSVttk reporter gene and iron oxide particles for PET imaging of cell viability and MR imaging of cell location. They applied cardiac ultrasound and electrocardiogram to validate its therapeutic potential for MI. The improved ventricular function was measured via ejection fraction and stroke volume. With increases in advanced technology on stem-cells based therapy, the evaluation of efficacy of MSCs engraftment will be more perfect and powerful.

## 5. Conclusion

In summary, the methods to induce BMSCs differentiation into cardiomyocyte-like cells include biochemical drug induction in vitro, such as 5-aza, BMP-2, AngII, DMSO, and various herbs. Chemical inducers are known to have the possible toxicity. Even under the best concentrations and optimal inducing time, chemical inducers may lead to cell death. Due to their toxicity and undesirable effect, chemical inducers are not able to be used in clinical translation. Furthermore, myocardial microenvironment could affect BMSCs differentiation. Thus, creating culture conditions that more closely mimic cardiac environment is a good idea, such as cardiomyocyte lysate, culture medium of myocardial cells. Therefore, differentiation methods with the myocardial microenvironment will be more prospective. A study [114] has shown that the inducing rate of culture medium of myocardial cells is not as good as CLM. The evidence indicated that some soluble substances contributing to inducing BMSCs differentiation could be released by cardiomyocytes. However, these substances could not be released until myocardial cells are broken [114, 115]. Because MSCs interact with cardiomyocytes by paracrine and autocrine after directly coculturing with cardiomyocytes, there are physical stimulations such as electrical activity and mechanical traction between MSCs and cardiomyocytes [68–70]. Thus, among various induction methods, CLM and direct coculturing with myocardial cells may be more feasible. It will be expected that application of both CLM and coculturing will further boost the MSCs differentiation into cardiomyocytes.

Human bone marrow mesenchymal stem cells have wide application prospect because it can be obtained autologously and have no immune rejection. Furthermore, it is easy to culture in vitro and can be induced to differentiate into myocardial cells through many ways. However, there are still several problems needed to be addressed. The specific pathways and the regulation mechanism of hMSCs differentiation into cardiomyocyte-like cells are still not clear. More studies are needed to determine optimal infusion dosage, timing with different induction method. To satisfy the clinical usage, it needs to ameliorate the conditions of induction and to further improve the differentiation efficiency. However, the clinical translation of stem-cell based therapy is a more complex process, and its efficacy needs to be fully investigated in a larger sample size and evaluated in a great quantity of preclinical experiments.

Therefore, what is needed in the stem cell research is the investigation of the best/safest cell type and improvement after clinical treatment for MI. The convincing researches need more considerations, well-conceived plan, and rigorous experiments. It is convinced that with the deepen of the research and the improvement of technology, the application perspective of BMSCs transplantation to treat MI will be extremely bright.

## Figures and Tables

**Table 1:** The inducer for BMSC differentiation.

Inducing condition	Year	Researcher	Culturing duration after induction	Detection marker
5-Aza	1995	Wakitani et al. [25]	24 h	7–10 days observing myotube-like structures and expressing cardiac-specific protein

	1999	Makino et al. [26]	24 h	1 week: myotube-like structures, 2 weeks: spontaneous beating, and 3 weeks: synchronous contraction, expressing cardiac-specific protein and exhibiting biological and electrophysiological characteristics of myocardial cells

BMP-2	2005	He Qizhi and Haijie [27]	24 h	The expression of Nkx2.5, GATA-4, cTnT, and CX43 increasing

Ang-II	2012	Xing et al. [28]	24 h	Expressing cTnI after 4 weeks, exhibiting morphological characteristics of myocardial cells, and being seen as muscle wire-like structures under the electron microscope

DMSO	1999	Skerjanc [29]	>6 d	Spontaneously beating cardiac myocytes after 6 days

Panax notoginseng saponins	2006	Yang et al. [30]	24 h	2 weeks: beginning to express MHC and more apparent after 4 weeks

Sal B	2007	Chen et al. [31]	24 h	Expression of NKX2.5 GATA-4 mRNA enhanced and peaked at 7 days; the expression of *α*-actin appeared at 14 days

Icariin	2008	Shao-Ying [32]	24 h	28 days, weakly expressing GATA-4, Nkx2-5, combining with 5-Aza enhance its induction

Astragaloside	2007	Xian et al. [33]	24 h, 48 h, and 72 h	4 weeks detecting cardiac-specific protein desmin, cTnI, *α*-MHC, and *β*-MHC, no significant difference in induction rate with different length

Microenvironment in vivo	2002	Toma et al. [34]		hMSCs could differentiate into myocardium and express myocardium specific protein in left ventricular microenvironment of SCID mice—cTnT and phosphoprotein regulating Ca-ATP activity at sarcoplasmic reticulum.

CLM	2005	Yuan et al. [35]	7 d	7 days, the cells growing well, expressing *α*-actin and cTnT

Coculturing with cardiomyocytes	2003	Rangappa et al. [36]	48 h	hMSCs coculturing with cardiomyocytes at a 1 : 1 ratio, expressing contractile proteins and cardiac specific genes, MHC, and beta-actin

## References

[1] Orlic D., Kajstura J., Chimenti S. (2001). Bone marrow cells regenerate infarcted myocardium. *Nature*.

[2] Friedenstein A. J., Chailakhyan R. K., Gerasimov U. V. (1987). Bone marrow osteogenic stem cells: in vitro cultivation and transplantation in diffusion chambers. *Cell and Tissue Kinetics*.

[3] Pittenger M. F., Mackay A. M., Beck S. C. (1999). Multilineage potential of adult human mesenchymal stem cells. *Science*.

[4] Soonpaa M. H., Koh G. Y., Klug M. G., Field L. J. (1994). Formation of nascent intercalated disks between grafted fetal cardiomyocytes and host myocardium. *Science*.

[5] Asahara T., Murohara T., Sullivan A. (1997). Isolation of putative progenitor endothelial cells for angiogenesis. *Science*.

[6] Beltrami A. P., Barlucchi L., Torella D. (2003). Adult cardiac stem cells are multipotent and support myocardial regeneration. *Cell*.

[7] Hu Z.-B., Zeng R., Guo W.-T., Lin H. (2008). Induction and differentiation of bone marrow mesenchymal stem cells. *Journal of Clinical Rehabilitative Tissue Engineering Research*.

[8] Erices A., Conget P., Minguell J. J. (2000). Mesenchymal progenitor cells in human umbilical cord blood. *British Journal of Haematology*.

[9] Lee O. K., Kuo T. K., Chen W.-M., Lee K.-D., Hsieh S.-L., Chen T.-H. (2004). Isolation of multipotent mesenchymal stem cells from umbilical cord blood. *Blood*.

[10] In't Anker P. S., Scherjon S. A., Kleijburg-Van Der Keur C. (2004). Isolation of mesenchymal stem cells of fetal or maternal origin from human placenta. *Stem Cells*.

[11] Yen B. L., Huang H.-I., Chien C.-C. (2005). Isolation of multipotent cells from human term placenta. *Stem Cells*.

[12] Kern S., Eichler H., Stoeve J., Klüter H., Bieback K. (2006). Comparative analysis of mesenchymal stem cells from bone marrow, umbilical cord blood, or adipose tissue. *Stem Cells*.

[13] Tropel P., Noël D., Platet N., Legrand P., Benabid A.-L., Berger F. (2004). Isolation and characterisation of mesenchymal stem cells from adult mouse bone marrow. *Experimental Cell Research*.

[14] Dominici M., le Blanc K., Mueller I. (2006). Minimal criteria for defining multipotent mesenchymal stromal cells. The International Society for Cellular Therapy position statement. *Cytotherapy*.

[15] Tocci A., Forte L. (2003). Mesenchymal stem cell: use and perspective. *Hematology Journal*.

[16] Dayoub H., Dumont R. J., Li J. Z. (2003). Human mesenchymal stem cells transduced with recombinant bone morphogenetic protein-9 adenovirus promote osteogenesis in rodents. *Tissue Engineering*.

[17] Jorgensen C., Djouad F., Apparailly F., Noël D. (2003). Engineering mesenchymal stem cells for immunotherapy. *Gene Therapy*.

[18] Kraitchman D. L., Heldman A. W., Atalar E. (2003). In vivo magnetic resonance imaging of mesenchymal stem cells in myocardial infarction. *Circulation*.

[19] Tuan R. S., Boland G., Tuli R. (2003). Adult mesenchymal stem cells and cell-based tissue engineering. *Arthritis Research & Therapy*.

[20] Jackson K. A., Majka S. M., Wang H. (2001). Regeneration of ischemic cardiac muscle and vascular endothelium by adult stem cells. *The Journal of Clinical Investigation*.

[21] Kocher A. A., Schuster M. D., Szabolcs M. J. (2001). Neovascularization of ischemic myocardium by human bone-marrow-derived angioblasts prevents cardiomyocyte apoptosis, reduces remodeling and improves cardiac function. *Nature Medicine*.

[22] Lodie T. A., Blickarz C. E., Devarakonda T. J. (2002). Systematic analysis of reportedly distinct populations of multipotent bone marrow-derived stem cells reveals a lack of distinction. *Tissue Engineering*.

[23] Gronthos S., Zannettino A. C. W., Hay S. J. (2003). Molecular and cellular characterisation of highly purified stromal stem cells derived from human bone marrow. *Journal of Cell Science*.

[24] Geng Y.-J. (2003). Molecular mechanisms for cardiovascular stem cell apoptosis and growth in the hearts with atherosclerotic coronary disease and ischemic heart failure. *Annals of the New York Academy of Sciences*.

[25] Wakitani S., Saito T., Caplan A. I. (1995). Myogenic cells derived from rat bone marrow mesenchymal stem cells exposed to 5-azacytidine. *Muscle & Nerve*.

[26] Makino S., Fukuda K., Miyoshi S. (1999). Cardiomyocytes can be generated from marrow stromal cells in vitro. *The Journal of Clinical Investigation*.

[27] He Qizhi T. Y., Haijie W. (2005). Effects of BMP-2 on differentiation of marrow-derived cardiac stem cells towards cardiomyocytes. *Acta Anatomica Sinica*.

[28] Xing Y., Lv A., Wang L., Yan X. (2012). The combination of angiotensin II and 5-azacytidine promotes cardiomyocyte differentiation of rat bone marrow mesenchymal stem cells. *Molecular and Cellular Biochemistry*.

[29] Skerjanc I. S. (1999). Cardiac and skeletal muscle development in P19 embryonal carcinoma cells. *Trends in Cardiovascular Medicine*.

[30] Yang Z. Q., Xian S. X., Wang C. H., Nan N. Y., Zhao L. C. (2006). Effects of panax notoginseng saponins on the differentiation of marrow mesenchymal stem cells into cardiomyocyte-like cells. *Traditional Chinese Drug Research & Clinical Pharmacology*.

[31] Chen J., Sun J.-C., Zou Y.-H. (2007). Differentiation of marrow mesenchymal stem cells into cardiomyocyte-like cells induced by salvianolic acid B. *Journal of the Fourth Military Medical University*.

[32] Shao-Ying L. (2008). Effects of icariin on bone marrow mesenchymal stem cell proliferation and differentiation into cardiomyocytes. *Journal of Beijing University of Traditional Chinese Medicine*.

[33] Xian S. X., Yang Z. Q., Wang C. H., Li N. Y., Zhao L. C. (2007). An experimental study on astragaloside inducing bone marrow mesenchymal stem cells to differentiate into cardiomyogenic cells in vitro. *Jouranl of Guangzhou Uiversity of Traditional Chinese Medicine*.

[34] Toma C., Pittenger M. F., Cahill K. S., Byrne B. J., Kessler P. D. (2002). Human mesenchymal stem cells differentiate to a cardiomyocyte phenotype in the adult murine heart. *Circulation*.

[35] Yuan Y., Chen L.-F., Zhang S.-Y., Wu W., Chen H., Yan X.-W. (2005). Differentiation of mesenchymal stem cells into cardio myogenic cells under the induction of myocardial cell lysate. *Chinese Journal of Cardiology*.

[36] Rangappa S., Entwistle J. W. C., Wechsler A. S., Kresh J. Y. (2003). Cardiomyocyte-mediated contact programs human mesenchymal stem cells to express cardiogenic phenotype. *Journal of Thoracic and Cardiovascular Surgery*.

[37] Taylor S. M. (1993). 5-aza-2′-deoxycytidine: cell differentiation and DNA methylation. *Leukemia*.

[38] Fukuda K. (2001). Development of regenerative cardiomyocytes from mesenchymal stem cells for cardiovascular tissue engineering. *Artificial Organs*.

[39] Jaquet K., Krause K., Stuts N., Zander A., Kuck K.-H. (2002). Azacytidine stimulated bone marrow derived human mesenchymal stem cells: percutaneous intramyocardial delivery. *Journal of the American College of Cardiology*.

[40] Huques S. (2002). Cardiac stem cells. *The Journal of Pathology*.

[41] Cai X., Nomura-Kitabayashi A., Cai W., Yan J., Christoffels V. M., Cai C.-L. (2011). Myocardial Tbx20 regulates early atrioventricular canal formation and endocardial epithelial-mesenchymal transition via Bmp2. *Developmental Biology*.

[42] de Pater E., Ciampricotti M., Priller F. (2012). Bmp signaling exerts opposite effects on cardiac differentiation. *Circulation Research*.

[43] Lee K.-H., Evans S., Ruan T. Y., Lassar A. B. (2004). SMAD-mediated modulation of YY1 activity regulates the BMP response and cardiac-specific expression of a GATA4/5/6-dependent chick Nkx2.5 enhancer. *Development*.

[44] Stottmann R. W., Choi M., Mishina Y., Meyers E. N., Klingensmith J. (2004). BMP receptor IA is required in mammalian neural crest cells for development of the cardiac outflow tract and ventricular myocardium. *Development*.

[45] Schneider M. D., Gaussin V., Lyons K. M. (2003). Tempting fate: BMP signals for cardiac morphogenesis. *Cytokine and Growth Factor Reviews*.

[46] Endtmann C., Ebrahimian T., Czech T. (2011). Angiotensin II impairs endothelial progenitor cell number and function in vitro and in vivo: implications for vascular regeneration. *Hypertension*.

[47] Xu J., Lin S. C., Chen J. (2011). CCR2 mediates the uptake of bone marrow-derived fibroblast precursors in angiotensin II-induced cardiac fibrosis. *The American Journal of Physiology—Heart and Circulatory Physiology*.

[48] Li L., Fan D., Wang C. (2011). Angiotensin II increases periostin expression via Ras/p38 MAPK/CREB and ERK1/2/TGF-beta1 pathways in cardiac fibroblasts. *Cardiovascular Research*.

[49] Kim Y. M., Jeon E. S., Kim M. R., Jho S. K., Ryu S. W., Kim J. H. (2008). Angiotensin II-induced differentiation of adipose tissue-derived mesenchymal stem cells to smooth muscle-like cells. *International Journal of Biochemistry and Cell Biology*.

[50] He J.-G., Chen S.-L., Huang Y.-Y., Chen Y.-L., Dong Y.-G., Ma H. (2010). The nonpeptide AVE0991 attenuates myocardial hypertrophy as induced by angiotensin II through downregulation of transforming growth factor-*β*1/Smad2 expression. *Heart and Vessels*.

[51] Wang N., Ren G.-D., Zhou Z. (2012). Cooperation of myocardin and smad2 in inducing differentiation of mesenchymal stem cells into smooth muscle cells. *IUBMB Life*.

[52] McBurney M. W., Jones-Villeneuve E. M. V., Edwards M. K. S., Anderson P. J. (1982). Control of muscle and neuronal differentiation in a cultured embryonal carcinoma cell line. *Nature*.

[53] van der Heyden M. A. G., van Kempen M. J. A., Tsuji Y., Rook M. B., Jongsma H. J., Opthof T. (2003). P19 embryonal carcinoma cells: a suitable model system for cardiac electrophysiological differentiation at the molecular and functional level. *Cardiovascular Research*.

[54] Ventura C., Maioli M. (2000). Opioid peptide gene expression primes cardiogenesis in embryonal pluripotent stem cells. *Circulation Research*.

[55] Skerjanc I. S., Petropoulos H., Ridgeway A. G., Wilton S. (1998). Myocyte enhancer factor 2C and Nkx2-5 up-regulate each other's expression and initiate cardiomyogenesis in P19 cells. *The Journal of Biological Chemistry*.

[56] Paquin J., Danalache B. A., Jankowski M., McCann S. M., Gutkowska J. (2002). Oxytocin induces differentiation of P19 embryonic stem cells to cardiomyocytes. *Proceedings of the National Academy of Sciences of the United States of America*.

[57] Rephaeli A., Aviram A., Rabizadeh E., Englender T., Shaklai M. (1990). The role of calcium in differentiation of leukemic cell lines. *Cancer Biochemistry Biophysics*.

[58] Sun J.-H. X.-Y., Huang W.-X., Yang Z.-Q. (2002). Advances in bone marrow mesenchymal stem cells induced to differentiate into cardiomyocytes under Chinese medicine intervention. *Traditional Chinese Drug Research & Clinical Pharmacology*.

[59] Li Z. X. S., Wang Z. (2007). Effect of panax notoginseng saponins on proliferation of bone marrow mesenchymal stem cells and their differentiation into cardiomyogenic cells. *Journal of Guangzhou University of Traditional Chinese Medicine*.

[60] Kong X. D., Qiu P., Wang Y. (2005). Differentiation of porcine marrow stromal cells into myocardium-like cells induced by Panax notoginosides in vitro. *Chinese Journal of Cardiovascular Review*.

[61] Wang N.-Y., Lu C.-J., Chen X.-H. (2005). Study on effect of ginsenoside Rg1 in promoting myocardiac vascular endothelial cell regeneration through induction on bone marrow stem cell's migration and differentiation in rabbits of myocardial infarction. *Chinese Journal of Integrated Traditional and Western Medicine*.

[62] Yang Q.-Y., Xian S.-X., Sun H.-R., Zhao L.-C., Wang C.-H. (2008). Experimental study on marrow mesenchyma stem cells differentiated into the cardic muscle type cells inducted by the medicine blood serum with Huangqi. *Liaoning Journal of Traditional Chinese Medicine*.

[63] Cao X.-X., Dai Y.-H., Zhang S.-Y. (2007). Differentiation of human mesenchymal stem cells into cardiomyocyte-like cells. *Basic & Clinical Medicine*.

[64] Jue X., Gong E. B. S., Qing D. (2006). Effect of microenvironment in vitro on the differentiation of bone marrow mesenchymal stem cells into cardiomyogenic cells. *Chinese Journal of Clinical Rehabilitation*.

[65] Wang X.-L., Wang J., Gong H.-B. (2008). Effects of myocardial microenvironment on BMSCs differentiating into cardiomyocyte-like cells. *Progress in Modern Biomedicine*.

[66] Li C.-M., Wang X.-L., Zhu H.-L., Wang J., Gong H.-B. (2013). Supernatant of myocardiocyte induces differentiation of bone marrow-derived mesenchymal stem cells. *Chinese Journal of Tissue Engineering Research*.

[67] He X.-Q., Chen M.-S., Li S.-H. (2010). Co-culture with cardiomyocytes enhanced the myogenic conversion of mesenchymal stromal cells in a dose-dependent manner. *Molecular and Cellular Biochemistry*.

[68] Xu M., Wani M., Dai Y.-S. (2004). Differentiation of bone marrow stromal cells into the cardiac phenotype requires intercellular communication with myocytes. *Circulation*.

[69] Plotnikov E. Y., Khryapenkova T. G., Vasileva A. K. (2008). Cell-to-cell cross-talk between mesenchymal stem cells and cardiomyocytes in co-culture. *Journal of Cellular and Molecular Medicine*.

[70] Cselenyák A., Pankotai E., Horváth E. M., Kiss L., Lacza Z. (2010). Mesenchymal stem cells rescue cardiomyoblasts from cell death in an in vitro ischemia model via direct cell-to-cell connections. *BMC Cell Biology*.

[71] Bodmer R. (1993). The gene tinman is required for specification of the heart and visceral muscles in *Drosophila*. *Development*.

[72] Tanaka M., Chen Z., Bartunkova S., Yamasaki N., Izumo S. (1999). The cardiac homeobox gene Csx/Nkx2.5 lies genetically upstream of multiple genes essential for heart development. *Development*.

[73] Pu W. T., Ishiwata T., Juraszek A. L., Ma Q., Izumo S. (2004). GATA4 is a dosage-sensitive regulator of cardiac morphogenesis. *Developmental Biology*.

[74] Gajewski K., Fossett N., Molkentin J. D., Schulz R. A. (1999). The zinc finger proteins pannier and GATA4 function as cardiogenic factors in Drosophila. *Development*.

[75] Horb M. E., Thomsen G. H. (1999). Tbx5 is essential for heart development. *Development*.

[76] Jamali M., Rogerson P. J., Wilton S., Skerjanc I. S. (2001). Nkx2-5 activity is essential for cardiomyogenesis. *The Journal of Biological Chemistry*.

[77] Monzen K., Zhu W., Kasai H. (2002). Dual effects of the homeobox transcription factor Csx/Nkx2-5 on cardiomyocytes. *Biochemical and Biophysical Research Communications*.

[78] Stennard F. A., Costa M. W., Elliott D. A. (2003). Cardiac T-box factor Tbx20 directly interacts with Nkx2-5, GATA4, and GATA5 in regulation of gene expression in the developing heart. *Developmental Biology*.

[79] Kinner B., Zaleskas J. M., Spector M. (2002). Regulation of smooth muscle actin expression and contraction in adult human mesenchymal stem cells. *Experimental Cell Research*.

[80] Chang K. S., Rothblum K. N., Schwartz R. J. (1985). The complete sequence of the chicken *α*-cardiac actin gene: a highly conserved vertebrate gene. *Nucleic Acids Research*.

[81] Mayer Y., Czosnek H., Zeelon P. E., Yaffe D., Nudel U. (1984). Expression of the genes coding for the skeletal muscle and cardiac actins in the heart. *Nucleic Acids Research*.

[82] Panteghini M. (2000). Present issues in the determination of troponins and other markers of cardiac damage. *Clinical Biochemistry*.

[83] Moscoso I., Centeno A., López E. (2005). Differentiation ‘in vitro’ of primary and immortalized porcine mesenchymal stem cells into cardiomyocytes for cell transplantation. *Transplantation Proceedings*.

[84] Qing-An L. (2011). Effect of ouabain on the expression of cell membrane Na^+^/k^+−^ATPase. *Journal of Xiaiming University*.

[85] Saunders R., Scheiner-Bobis G. (2004). Ouabain stimulates endothelin release and expression in human endothelial cells without inhibiting the sodium pump. *European Journal of Biochemistry*.

[86] Boengler K., Schulz R., Heusch G. (2006). Connexin 43 signalling and cardioprotection. *Heart*.

[87] Maltsev V. A., Wobus A. M., Rohwedel J., Bader M., Hescheler J. (1994). Cardiomyocytes differentiated in vitro from embryonic stem cells developmentally express cardiac-specific genes and ionic currents. *Circulation Research*.

[88] Zhang Y. M., Hartzell C., Narlow M., Dudley S. C. (2002). Stem cell-derived cardiomyocytes demonstrate arrhythmic potential. *Circulation*.

[89] Viatchenko-Karpinski S., Fleischmann B. K., Liu Q. (1999). Intracellular Ca^2+^ oscillations drive spontaneous contractions in cardiomyocytes during early development. *Proceedings of the National Academy of Sciences of the United States of America*.

[90] Maltsev V. A., Rohwedel J., Hescheler J., Wobus A. M. (1993). Embryonic stem cells differentiate in vitro into cardiomyocytes representing sinusnodal, atrial and ventricular cell types. *Mechanisms of Development*.

[91] Strauer B. E., Brehm M., Zeus T. (2001). Intracoronary, human autologous stem cell transplantation for myocardial regeneration following myocardial infarction. *Deutsche Medizinische Wochenschrift*.

[92] Schächinger V., Erbs S., Elsässer A. (2006). Intracoronary bone marrow-derived progenitor cells in acute myocardial infarction. *The New England Journal of Medicine*.

[93] Kang H.-J., Lee H.-Y., Na S.-H. (2006). Differential effect of intracoronary infusion of mobilized peripheral blood stem cells by granulocyte colony-stimulating factor on left ventricular function and remodeling in patients with acute myocardial infarction versus old myocardial infarction: the MAGIC cell-3-DES randomized, controlled trial. *Circulation*.

[94] Meyer G. P., Wollert K. C., Lotz J. (2006). Intracoronary bone marrow cell transfer after myocardial infarction: eighteen months' follow-up data from the randomized, controlled BOOST (Bone marrow transfer to enhance ST-elevation infarct regeneration) trial. *Circulation*.

[95] Tse H. F., Yiu K. H., Lau C. P. (2007). Bone marrow stem cell therapy for myocardial angiogenesis. *Current Vascular Pharmacology*.

[96] Hu X., Wang J., Chen J. (2007). Optimal temporal delivery of bone marrow mesenchymal stem cells in rats with myocardial infarction. *European Journal of Cardio-Thoracic Surgery*.

[97] Janssens S., Dubois C., Bogaert J. (2006). Autologous bone marrow-derived stem-cell transfer in patients with ST-segment elevation myocardial infarction: double-blind, randomised controlled trial. *The Lancet*.

[98] Ghostine S., Carrion C., Souza L. C. G. (2002). Long-term efficacy of myoblast transplantation on regional structure and function after myocardial infarction. *Circulation*.

[99] Fukushima S., Coppen S. R., Lee J. (2008). Choice of cell-delivery route for skeletal myoblast transplantation for treating post-infarction chronic heart failure in rat. *PLoS ONE*.

[100] Widimsky P., Penicka M., Lang O. (2006). Intracoronary transplantation of bone marrow stem cells: background, techniques, and limitations. *European Heart Journal Supplements*.

[101] Mozid A. M., Arnous S., Sammut E. C., Mathur A. (2011). Stem cell therapy for heart diseases. *British Medical Bulletin*.

[102] Vicario J., Piva J., Pierini A. (2002). Transcoronary sinus delivery of autologous bone marrow and angiogenesis in pig models with myocardial injury. *Cardiovascular Radiation Medicine*.

[103] Yokoyama S.-I., Fukuda N., Li Y. (2006). A strategy of retrograde injection of bone marrow mononuclear cells into the myocardium for the treatment of ischemic heart disease. *Journal of Molecular and Cellular Cardiology*.

[104] Dib N., Menasche P., Bartunek J. J. (2010). Recommendations for successful training on methods of delivery of biologics for cardiac regeneration: a report of the International Society for Cardiovascular Translational Research. *JACC: Cardiovascular Interventions*.

[105] Patel A. N., Geffner L., Vina R. F. (2005). Surgical treatment for congestive heart failure with autologous adult stem cell transplantation: a prospective randomized study. *The Journal of Thoracic and Cardiovascular Surgery*.

[106] Hamano K., Nishida M., Hirata K. (2001). Local implantation of autologous bone marrow cells for therapeutic angiogenesis in patients with ischemic heart disease—clinical trial and preliminary results. *Japanese Circulation Journal*.

[107] Hagège A. A., Marolleau J.-P., Vilquin J.-T. (2006). Skeletal myoblast transplantation in ischemic heart failure: Long-term follow-up of the first phase I cohort of patients. *Circulation*.

[108] Halkos M. E., Zhao Z.-Q., Kerendi F. (2008). Intravenous infusion of mesenchymal stem cells enhances regional perfusion and improves ventricular function in a porcine model of myocardial infarction. *Basic Research in Cardiology*.

[109] Britten M. B., Abolmaali N. D., Assmus B. (2003). Infarct remodeling after intracoronary progenitor cell treatment in patients with acute myocardial infarction (TOPCARE-AMI): mechanistic insights from serial contrast-enhanced magnetic resonance imaging. *Circulation*.

[110] Bel A., Planat-Bernard V., Saito A. (2010). Composite cell sheets: a further step toward safe and effective myocardial regeneration by cardiac progenitors derived from embryonic stem cells. *Circulation*.

[111] Miyahara Y., Nagaya N., Kataoka M. (2006). Monolayered mesenchymal stem cells repair scarred myocardium after myocardial infarction. *Nature Medicine*.

[112] Hou D., Youssef E. A.-S., Brinton T. J. (2005). Radiolabeled cell distribution after intramyocardial, intracoronary, and interstitial retrograde coronary venous delivery: implications for current clinical trials. *Circulation*.

[113] Lee A. S., Xu D., Plews J. R. (2011). Preclinical derivation and imaging of autologously transplanted canine induced pluripotent stem cells. *The Journal of Biological Chemistry*.

[114] Wang J. S., Shum-Tim D., Galipeau J., Chedrawy E., Eliopoulos N., Chiu R. C.-J. (2000). Marrow stromal cells for cellular cardiomyoplasty: feasibility and potential clinical advantages. *Journal of Thoracic and Cardiovascular Surgery*.

[115] Murry C. E., Soonpaa M. H., Reinecke H. (2004). Haematopoietic stem cells do not transdifferentiate into cardiac myocytes in myocardial infarcts. *Nature*.

